# The efficacy and safety of urethral injection therapy for urinary incontinence in women: a systematic review

**DOI:** 10.6061/clinics/2016(02)08

**Published:** 2016-02

**Authors:** Priscila Katsumi Matsuoka, Rafael Fagionato Locali, Aparecida Maria Pacetta, Edmund Chada Baracat, Jorge Milhem Haddad

**Affiliations:** I Hospital das Clínicas da Faculdade de Medicina da Universidade de São Paulo (HC-FMUSP), Departamento de Obstetrícia e Ginecologia, Disciplina de Ginecologia, Divisão de Uroginecologia, São Paulo/SP, Brazil; II Hospital das Clínicas da Faculdade de Medicina da Universidade de São Paulo (HC-FMUSP), Departamento de Cirurgia, Disciplina de Urologia, São Paulo/, SP, Brazil; III Hospital das Clínicas da Faculdade de Medicina da Universidade de São Paulo (HC-FMUSP), Departamento de Obstetrícia e Ginecologia, Disciplina de Ginecologia, São Paulo/, SP, Brazil

**Keywords:** Bulking Agents, Injections, Intraurethral Injections, Urinary Incontinence

## Abstract

To evaluate the efficacy and safety of different bulking agents for treating urinary incontinence in women, a systematic review including only randomized controlled trials was performed. The subjects were women with urinary incontinence. The primary outcomes were clinical and urodynamic parameters. The results were presented as a weighted mean difference for non-continuous variables and as relative risk for continuous variables, both with 95% confidence intervals. Initially, 942 studies were identified. However, only fourteen eligible trials fulfilled the prerequisites. Altogether, the review included 1814 patients in trials of eight different types of bulking agents, and all studies were described and analyzed. The measured outcomes were evaluated using a large variety of instruments. The most common complications of the bulking agents were urinary retention and urinary tract infection. Additionally, there were certain major complications, such as one case of death after use of autologous fat. However, the lack of adequate studies, the heterogeneous populations studied, the wide variety of materials used and the lack of long-term follow-up limit guidance of practice. To determine which substance is the most suitable, there is a need for more randomized clinical trials that compare existing bulking agents based on standardized clinical outcomes.

## INTRODUCTION

Urinary incontinence is defined as the complaint of involuntary leakage of urine 1. This condition clinically presents as urgency incontinence, stress urinary incontinence, or mixed urinary incontinence, although stress urinary incontinence is the most prevalent type in women.

There is a wide variety of both clinical and surgical treatment approaches for incontinence. However, despite the large number of surgical procedures available to treat urinary incontinence, not all patients can benefit from the surgery because the procedure-related risks (associated with the patients' clinical comorbidities) may outweigh the benefits of this therapy. Furthermore, certain patients and especially those with intrinsic sphincter deficiency and a fixed urethra, are difficult to treat, showing poor results when subjected to conventional surgery for urinary incontinence 2,3,4,5. For those patients who present treatment failure, there are very few alternatives to treat stress urinary incontinence. Bulking agents are one option, offering less invasive augmentation of the urethra than sling procedures and artificial sphincters 4,5.

For over a century, injection of urethral bulking agents has been used in the treatment of urinary incontinence. Over the past 50 years, this treatment modality has become more popular, particularly due to the development of new materials. In addition, this therapy deserves special attention because it is a minimally invasive treatment with rapid recovery and a low morbidity rate 6.

Regardless of the bulking agent used, the mechanism of action of urethral injections involves reduction of the inner diameter of the urethra. Thus, the final effect is coaptation of the urethral lumen, which can lead to increased urethral resistance and improvement in urinary incontinence.

Several agents have been used for urethral bulking. However, in terms of efficacy and safety, no clear conclusions can be drawn from trials to date to establish the best agent for this treatment 7,8. Therefore, the aim of the present study was to evaluate the efficacy and safety of the different bulking agents used in urethral injection therapy for urinary incontinence in women.

## METHODS

### Inclusion and exclusion criteria

#### Study selection

Randomized clinical trials were included. Prospective, observational, cohort and case studies; letters; reviews; and animal studies were excluded.

#### Patients

We selected studies analyzing adult female patients with a diagnosis of urinary incontinence based on clinical complaint and urodynamic investigation. There were no restrictions on patients' ethnicity or type of stress urinary incontinence.

#### Intervention

We analyzed randomized clinical trials that evaluated urethral injection techniques for the treatment of urinary incontinence. These studies had to have compared techniques for urethral bulking with each other or with a different treatment modality, regardless of the type and concentration of agents used and of the duration and frequency of treatment. All studies assessing different treatment techniques that did not include urethral bulking or that switched interventions between groups during the course of the study were excluded from our analysis. Studies using stem cells or urethral injection after surgery for cancer treatment or sex change were also excluded.

#### Clinical outcomes

The primary outcomes were divided into clinical and urodynamic parameters. Clinical parameters included symptom improvement (assessed by subjective questions about clinical improvement), 24-hour pad test results after the procedure, the impact on quality of life (assessed by the King's College Hospital Quality of Health Questionnaire), voiding diary results and procedure-related pain (assessed by 10-point Likert-type visual pain scales). Regarding objective parameters, residual volume and other data obtained from urodynamic investigation were evaluated.

We did not stratify these parameters according to the assessment time points described in the primary study. In studies investigating the outcome at several time points, we considered only data referring to the last assessment period.

#### Literature search strategy

A systematic literature search was conducted using MEDLINE, Embase, Cochrane and LILACS, including articles published until April 4^th^, 2015. For this purpose, we used a search strategy with high sensitivity and low specificity, based on keywords and synonyms for urinary incontinence, bulking agents, injections, and periurethral injections, without limits regarding study design, dates, or country of origin. This strategy was adjusted according to the search engine used by each database.

### Standardization of literature review

#### Study selection

All articles retrieved from each database were initially screened based on information found in the title and abstract and were grouped as selected or non-selected studies. This distribution aimed at initial selection of studies that could potentially be included. Articles that did not have sufficient information in the title or abstract to allow us to define a category were read in full and were subsequently also classified as selected or non-selected studies.

All selected studies were reviewed and read in full. After assessment of methodological quality, the articles were either included or excluded from our study according to the previously determined inclusion criteria. Moreover, the references of all selected studies were analyzed to increase the sensitivity of the systematic review. For the same purpose, all related articles were also reviewed.

Two researchers independently performed the process of screening the studies. At the end of this stage, the researchers compared the selected studies, and any discrepancies were resolved by consensus.

#### Assessment of methodological quality

All selected studies were assessed for methodological quality by two researchers according to the technique developed by Jadad et al. in 1996 9. Methodological quality assessment was not used as an inclusion or exclusion criterion, but rather as a predictor of the strength of the evidence provided by an individual study.

#### Statistical analysis

Non-continuous variables are expressed as the weighted mean difference and continuous variables are expressed as the odds ratio, both followed by their respective 95% confidence intervals (CIs). The level of significance was set at 5% (rejection of the null hypothesis at the 0.05 level).

## RESULTS

A total of 942 studies were identified by the search strategy. This included articles retrieved from each database searched, related articles, and references obtained from the selected studies. Of these articles, 802 were retrieved from MEDLINE/PubMed; 137, from Embase; and 3, from LILACS.

**Figure 1:**
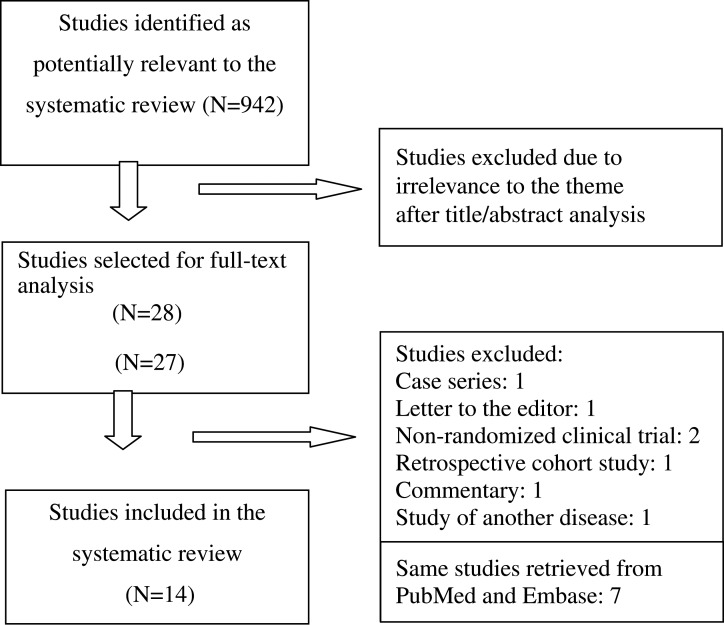
Search strategies used to identify studies.

After the initial screening, 28 studies were selected to be read in full. However, only fourteen eligible trials fulfilled the prerequisites for the systematic review. The flow chart of the study selection is shown in Figure 1.

The included studies showed significant methodological heterogeneity, with a broad range of treatment proposals (Table 1). The methodological characteristics of the selected studies and their main biases are summarized in Table 2.

In this review, 1814 patients were included. Altogether, the review included fourteen trials of seven different types of intraurethral injection: glutaraldehyde cross-linked collagen (Contigen™), a porcine dermal implant (Permacol™), solid silicone elastomer (Macroplastique™), autologous fat, pyrolytic carbon (Durasphere™), calcium hydroxyapatite (Coaptite™), hydrogel (Bulkamid™) and dextran copolymer (Zuidex™). The first trial compared solid silicone with the porcine dermal implant and showed an improvement of at least 1 Stamey grade at 12 months (*p*<0.001) in favor of silicone. Stamey grading is specifically a 4-level scale of incontinence severity, ranging from 0 (continent/dry) to 3 (total incontinence, regardless of activity) 12. Nevertheless, for the other outcome measurements, such as quality of life, pad test results, and patient and physician assessment, there was no difference between silicone and porcine dermal implant efficacy 12. Another trial compared autologous fat with placebo and did not show a significant difference in efficacy 14. Moreover, one patient died because of pulmonary embolism due to the autologous fat injection 15.

A study by Corcos et al. 7 compared collagen injections with three surgical techniques. When using intent-to-treat analysis, the success rate among patients treated with surgery was slightly higher than the rate in the collagen group, with a non-significant difference of -3.71% (*p*=0.334) 7. Use of pyrolytic carbon was shown to be as effective as collagen injections after 1 year of follow-up, and the required substance volume was significantly lower using pyrolytic carbon 4. Furthermore, there was no difference in Stamey grade with the use of calcium hydroxyapatite compared with collagen 17. In 2009, Lightner et al. 16 also compared dextran copolymer with collagen injections and the results at 12 months showed a smaller proportion of women with no response in terms of urinary leakage upon provocation testing using dextran copolymer 16.

Moreover, Kuhn et al. 11 tested collagen injections at two different anatomical sites. Continence was found in 66.6% of patients in the midurethral group and in 60% in the bladder neck group. Both midurethral and bladder neck collagen injections improved patient satisfaction nearly equally, with a small advantage for midurethral injections 10.

A trial carried out by Meulen et al. 14 compared silicone injections with pelvic floor muscle exercises in patients who had failed previous conservative treatment. Despite the fact that in terms of subjective parameters, there was a significantly greater increase in the incontinence-related Quality of Life Questionnaire score in the silicone group compared with the control group (*p*=0.017), in terms of objective parameters, there was no difference between the groups. At the 3^rd^ month, the results of the pad test showed improvement, although without a significant difference between the two groups 14. A study by Ghoniem et al. 13 showed that silicone injection was significantly more effective than collagen injections for the treatment of stress urinary incontinence, primarily due to intrinsic sphincter deficiency, with a 2.1% cure rate difference. A study by Andersen 19 evaluated the clinical success of pyrolytic carbon compared with collagen in the treatment of stress urinary incontinence caused by intrinsic sphincter deficiency and the results showed that 40% of pyrolytic carbon-treated patients and 14% of collagen-treated patients were dry. Moreover, a trial by Anders et al. 18 compared silicone with collagen in patients with intrinsic sphincter deficiency, and there was no difference in subjective or objective parameters between the groups.

A study comparing dextran copolymer injected via the periurethral and transurethral routes considered patients as dry only if they had 100% improvement and no leakage episodes 10. The mean improvement was not significantly different between the two groups at the 3^rd^, 6^th^, and 12^th^ months of follow-up 10, although postoperative urinary retention was significantly higher in the periurethral injection group (*p*<0.05) 10.

Finally, the clinical trial comparing collagen with polyacrylamide hydrogel (Bulkamid) showed a 50% or greater decrease in leakage and urinary incontinence episodes at 12 months in both groups, but not between groups 27. At 12 months of follow-up, the responder rate (subjective improvement or cure) was 77.1% (145 of 188 women) in the hydrogel group and 70.0% (70 of 100) in the collagen group (*p*=0.201).

The main results of the studies and the adverse effects of the bulking agents are summarized in Table 2. The measured outcomes were evaluated using a large variety of instruments and the outcomes in the studies of silicone compared with collagen were measured at different times (at 6 months in one study, at 12 months in the second study, and at 5 years in the third study). Therefore, we could not perform a meta-analysis with common variables of the studies.

## DISCUSSION

Urinary incontinence is a highly prevalent comorbidity among women, with a significant negative impact on quality of life; thus, effective treatment is imperative 2. It is known that due to associated comorbidities, many women are at high surgical risk. In this context, injecting bulking agents to treat stress urinary incontinence seems to be an attractive concept. Most procedures can be performed using regional or local anesthesia and even on an outpatient basis and the procedure is minimally invasive 7,20.

There is a large and considerably varied body of literature on this treatment technique. Several bulking agents have been reported, such as Teflon, autologous fat, silicone micro-implants, pyrolytic carbon, dextran polymer, collagen, polyacrylamide hydrogel and porcine dermal implants. However, there is no consensus as to the best agent and injection technique, so an accurate systematic review on the subject is warranted to evaluate existing information and identify the most suitable approach for patients.

Although systematic reviews are classified as secondary studies, they provide the best level of evidence to identify and support approaches used in health interventions. Here, we elaborated upon a systematic review including only randomized clinical trials and extensively analyzed the studies' methodological quality.

The analysis varied from study to study. A study by Schulz showed that neither objective (pad test results) nor subjective outcome mean improvement in 12 months was significantly different between the two standard routes of bulking agent injection. However, there was more urinary retention following injection via the transurethral route. Moreover, the site of collagen injection, whether midurethral or in the bladder neck, resulted in no difference in patient satisfaction, with both groups achieving a high grade of satisfaction within 10 months.

There are more data on collagen in the literature and studies on this substance showed significant improvement in the Stamey score but did not show differences in parameters such as quality of life and pad test results.

A systematic review by Ghoniem and Miller 22 included studies only on Macroplastique from 1990-2010. The authors specifically performed a meta-analysis of clinical trials and prospective, observational and cohort studies reporting treatment outcomes 22. In contrast, in the present review, we included only randomized clinical trials with the highest level of evidence, without limitation to year of publication. Despite these inclusion criteria, we noticed methodological quality heterogeneity among the included studies, as showed by the Jadad score, ranging from 2-4.

Comparing our review with a Cochrane Library review performed by Kirchin et al., we can highlight that in their study, quasi-randomized and randomized trials until May 2011 were included, whereas in ours, only randomized clinical trials until April 2015 were included 28. Moreover, the Cochrane review did not include the LILACS or Embase database. According to our inclusion criteria, we excluded studies using stem cells because the results of stem cell therapy for urinary incontinence are not well established in the literature, so the comparison would not be precise.

Our comparison of study results was hindered because of the absence of a standardized method to assess patients' symptoms and the lack of a standardized concept of a successful surgical procedure. In addition, this review selected fourteen studies with seven different types of bulking agents, thus preventing us from drawing conclusions that can be extrapolated to clinical practice. Although there are currently good-quality studies, methodological heterogeneity in the measurement of clinical outcomes in primary studies leads to impossibility in comparing results, which in turn hinders performance of a systematic meta-analysis on the topic 23.

Therefore, we suggest that more randomized clinical trials studying new substances that have already shown promising results in non-randomized prospective studies should be performed 24,25. To reduce bias, the studies should have the same patient inclusion criteria and should exclude patients with mixed urinary incontinence. The outcome measure should include patients' subjective evaluation of symptoms of urinary incontinence using already-existent questionnaires to evaluate urinary incontinence and pad test results. It is important to highlight that although the Stamey scoring system has its limitations, many studies have used this score.

The literature lacks studies with long-term follow-up of bulking agents. In the present review, only three studies had a follow-up longer than two years. There was a tendency for the improvement in urinary incontinence to decrease over time in these studies. Beyond that, the short-term analysis from the studies does not give much information about repeated injections or about the cost-effectiveness aspects of both first and repeated treatments.

Based on the studies included in this systematic review, we can say that the majority of bulking agents are safe, mainly because they did not show major adverse effects, except for the study using autologous fat, which had one death. However, not all adverse events were covered because we did not include case series with long-term follow-up, as reported for Deflux 26; this study demonstrated that the material itself was associated with more frequent pseudoabscess and *de novo* urge incontinence.

The lack of adequate studies, the heterogeneous populations studied, the wide variety of materials used and the lack of long-term follow-up limit guidance of practice. To determine which substance is the most suitable, there is a need for more randomized clinical trials that compare existing bulking agents based on standardized clinical outcomes.

## AUTHOR CONTRIBUTIONS

Matsuoka PK and Haddad JM were responsible for the project development, data collection, manuscript writing and meta-analysis. Locali RF was responsible for the manuscript writing and meta-analysis. Pacetta AM and Baracat EC were responsible for the manuscript editing.

## Figures and Tables

**Table 1:** Summary of the selected studies and their methodological quality.

No.	Authors	Intervention (n)	Control (n)	Type of UI	Primary outcome(s)	Secondary outcome(s)	Follow-up	Adverse effect(s)	Criteria for ISD
1	Schulz et al. (2004) 10	Periurethral injection with dextran copolymer (20)	Transurethral injection with dextran copolymer (20)	SUI (n=36)MUI (n=4)	Patients dry only if they had 100% improvement and no leakage episodes	Subjective mean improvement	1, 3, 6 and 12 months	Urinary retention	MUCP <20 cmH_2_O and a VLPP <60 cmH_2_O
2	Kuhn et al. (2008) 11	Midurethral injection with collagen TI (15)	Bladder neck injection with collagen TI (15)	SUI (n=30)	Patient satisfaction (VAS)	Cough test, urethral resting pressure, functional urethral length	6 weeks and 10 months	Residual urine (US)	NM
3	Bano et al. (2005) 12	Silicone TI (24)	Porcine dermal implant (27) [TI (13)+ PI (14)]	SUI (n=50)	Pad test	Stamey, KCQ	6 weeks and6 months	Urinary retention, urge incontinence	NM
4	Ghoniem et al. (2009) 13	Silicone TI (122)	Collagen TI (125)	SUI with ISD (n=247)	Stamey	I-QOL, pad test	1, 3, 6 and 12 months	Genitourinary adverse effects	NM
5	Meulen et al. (2009) 14	Silicone TI (24)	Pelvic floor muscle exercises (21)	SUI with urethral hypermobility (n=45)	Pad test Stamey	Patient global impression, I-QOL	3 and 12 months	Genitourinary adverse effects, leakage implant	NA
6	Lee et al. (2001) 15	Autologous fat PI (35)	Placebo PI (33)	SUI (n=68)	Pad test	UIQ, MUCP, LPP	1, 2, 3, 6, 9, 12, 18 and 24 months	UTI, liposuction site infection, urinary retention, fat embolism	NM
7	Corcos et al. (2005) 7	Collagen TI (64)	Urinary incontinence surgery* (54)	MUI or SUI (n=133)	Pad test	Symptoms of incontinence, general QOL, disease-specific QOL, and depression	1, 3, 6 and 12 months	Urinary retention, transient hematuria, urinary infection, voiding difficulty	NA
8	Lightner et al. (2001) 4	Pyrolytic carbon TI (178)	Collagen TI (177)	SUI with ISD (n=355)	Pad test Stamey	Adverse effects	1, 3, 6 and 12 months	Urinary retention, urgency	History and VLPP <90 cmH_2_O
9	Lightner et al. (2009) 16	Dextran polymer (143)	Collagen (88)	SUI (n=344)	The proportion of women who achieved a ≥50% reduction in urinary leakage on provocation testing performed at baseline presentation com- pared with that at 12 months after the last treatment	Stamey, pad test, voiding diary, I-QOL	1, 2, 3, 6 and 12 months	Urinary retention, urgency, Urinary infection, pain	VLPP <100 cmH_2_O
10	Mayer et al. (2007) 17	Calcium hydroxyapatite (158) [TI (145)+ PI (13)]	Collagen (138) [TI (123)+ PI (15)]	SUI with ISD (n=296)	Stamey	QOL, pad test	12 months	NM	NM
11	Anders et al. (1999) 18	Silicone (34)	Collagen (26)	SUI with ISD (n=60)	Patient satisfaction, KCQ	Symptoms of incontinence, frequency, urgency, pad test	60 months	NM	NM
12	Andersen (2002) 19	Pyrolytic carbon (26)	Collagen (26)	SUI with ISD (n=52)	Stamey	Pad test	2.6 and 2.8 years	NM	VLPP <90 cmH_2_O
13	Maher et al. (2005) 20	Silicone TI (23)	Pubovaginal sling (22)	SUI with ISD (n=45)	Success rates, complications, costs	SUDI, IIQ, pad test	6 weeks and 6, 12, and 60 months	Frequency, nocturia, urgency, urge incontinence, stress incontinence, voiding difficulty	MUCP <20 cmH_2_O
14	Sokol et al. (2014) 27	Polyacrylamide hydrogel (229)	Collagen (116)	SUI or stress-predominant mixed UI	The proportion of women at the 12-month follow-up with a ≥50% decrease from baseline in leakage, as measured by the 24-hour pad test, and a ≥50% decrease from baseline in the self-reported daily number of incontinence episodes	The proportion of women dry (4 gm or less) or improved according to the 24-hour pad test, ICIQ-UI, patient diary, responder rate, I-QOL	1, 3, 6, 9 and 12 months	Urinary retention, *de novo* urge incontinence, dysuria, excreted bulking material, hematuria, nocturia non-acute urinary pain,urinary tract infection	VLPP <100 cmH_2_O

**<?ENTCHAR ast?>:** Surgeries for urinary incontinence included Burch surgery, sling procedures, and bladder neck suspension. UI - urinary incontinence; MUI - mixed urinary incontinence; SUI - stress urinary incontinence; TI - transurethral injection; VAS - visual analog scale; NM - not mentioned; PI - periurethral injection; KCQ - King's College Hospital Quality of Health Questionnaire; Stamey - Stamey scoring system; I-QOL - Urinary Incontinence Quality of Life Scale; NA - not applicable; UIQ - Urinary Incontinence Questionnaire; MUCP - maximal urethral closure pressure; LPP - leak point pressure; SUDI - Short Urinary Distress Inventory; IIQ - Incontinence Impact Questionnaire; ISD - intrinsic sphincteric deficiency; ICIQ-UI - International Consultation on Incontinence Modular Questionnaire-Urinary Incontinence Form.

**Table 2:** Main results of the selected studies and their main adverse effects.

No.	Authors	Bulking agent	Previous treatment	Results – Primary outcome	Results – Secondary outcome	Main adverse effects	Necessity of reinjection
1	Schulz et al. (2004) 10	Dextran copolymer	Conservative treatments*	Dry: PI 1/17 and TI 3/17*p*=NS12 months	Subjective mean improvement: PI 37% in 6/17 and TI 36% in 7/17*p*=NS12 months	Urinary retention: PI 6/20 and TI 1/20 *p*<0.05	Yes, but number ND
2	Kuhn et al. (2008) 11	Collagen	NM	Patient satisfaction (VAS): bladder neck (BN): median of 8 (95% CI 5-9); midurethral (MU): 8 (95% CI 7-10)*p*<0.0510 months	MUCP: BN: increase, *p*<0.05; MU: increase, *p*<0.05postoperative	Urinary retention: BN 1/15 and MU 4/15	ND
3	Bano et al. (2005) 12	Silicone and PDI	PDI: sling (3) colposuspension (4); silicone: sling (2) and (1)	Pad test: dry: silicone 15/24 and PDI 9/246 months	Stamey improvement: silicone 14/24 and PDI 10/24 at 6 monthsKCQ: silicone 14/24 and PDI 7/24 at 6 months	Urinary retention: silicone 2/25 and PDI 1/25	ND
4	Ghoniem et al. (2009) 13	Silicone and collagen	Conservative treatments*	Stamey improvement: silicone 75/122 and collagen 60/125*p*<0.0512 months	I-QOL: NS difference after treatmentPad test: NS difference after treatment	UTI: silicone 29/122 and collagen 31/125Pyelonephritis: silicone 1/125	ND
5	ter Meulen et al. (2009) 14	Silicone *vs*. PFME	Conservative treatments*	Pad test: NS difference after treatment at 3 monthsStamey: silicone: improvement at 3 months, *p*<0.05	I-QOL: silicone showed better results at 3 months, *p*<0.05	Urinary retention: silicone 19/24Leakage of implant: 2/24	5 ml silicone administered in 2 women after the 3 months
6	Lee et al. (2001) 15	Autologous fat vs. placebo (saline injection)	19 in each group received conservative treatments*	Pad test: NS difference after treatment for 3 months	MUCP and LPP: NS difference after treatment for 3 months	One death due to pulmonary fat embolismUrinary retention: fat 6/35 and saline 0/33UTI: fat 6/35 and saline 3/33	3 injections in the control group; in the fat group 1/27 women received 2 injections, and 26 received 3 injections
7	Corcos et al. (2005) 7	Collagen *vs*. surgery	NM	Pad test: NS difference after 12 months	SF-36 and I-QOL: NS difference after 12 months	Urinary retention: collagen 11/20 and surgery 9/45UTI: collagen 0/20 and surgery 4/45	3 injections (interval of 1 month)
8	Lightner et al. (2001) 4	Pyrolytic carbon *vs*. collagen	Conservative treatment or anti-incontinence surgical procedures that failed	Stamey improvement: pyrolytic carbon 49/61 and collagen 47/6812 months	--------	Increased incidence of urinary retention in the pyrolytic carbon group (16.9% *vs*. 3.4%)*p*=0.001	Maximum of 5 injections
9	Lightner et al. (2009) 16	Dextran polymer *vs*. collagen	Conservative treatments*	Dextran polymer was not equivalent to collagen in 12 months	Stamey, pad test, voiding diary, I-QOL Dextran polymer was not equivalent to collagen in 12 months	Urinary retention: 28% in the dextran polymer group	Maximum of 3 injections
10	Mayer et al. (2007) 17	CaHA *vs*. collagen	Conservative treatments*	Stamey: same efficacy for CaHA and collagen at 12 months	QOL significant at 12 months	Urinary retention: CaHA 52/158 and collagen 45/138	Maximum of 3 injections
11	Anders et al. (1999) 18	Silicone *vs*. collagen	UI surgery	KCQ: NS difference after 60 months	----------	ND	ND
12	Andersen (2002) 19	Pyrolytic carbon (PC) *vs*. collagen	ND	Stamey improvement: PC 20/26 in 2.6 years and collagen 16/26 in 2.8 years	Pad test: dry PC 10/25 and collagen 3/21	ND	ND
13	Maher et al. (2005) 20	Silicone *vs*. sling	Conservative treatments*	Symptomatic and patient satisfaction success greater in the sling group at 6 months (*p*<0.001)	Pad test, SUDI and IIQ: NS difference after 6 months	Voiding dysfunction: silicone 1/23 and sling 4/22Urge incontinence: silicone 7/14 and sling 4/13	5/22 required a top-up transurethral injection
14	Sokol et al. (2014) 27	Polyacrylamide hydrogel *vs*. collagen	Conservative treatments*	Hydrogel demonstrated non-inferiority (*p*<0.003) but not superiority (*p*=NS) to collagen gel for the primary efficacy end point (ITT)	ICIQ-UI and I-QOL results showed considerable improvement in each treatment group, but not between the groups	Implantation site pain: collagen 9/116 and hydrogel 28/229	Up to 3 injections

CaHA - calcium hydroxyapatite; IIQ - Incontinence Impact Questionnaire; ISD - intrinsic sphincter deficiency; KCQ - King's College Hospital Quality of Health Questionnaire; MUCP - maximum urethral closure pressure; MUI - mixed urinary incontinence; NS - not significant; PDI - porcine dermal implant; PFME - pelvic floor muscle exercises; SF-36 - 36-item Short-Form Health Survey; SUDI - Short Urinary Distress Inventory; SUI - stress urinary incontinence; I-QOL - Urinary Incontinence Quality of Life Scale; VLPP - Valsalva leak point pressure; ND - not described; * including hormone replacement therapy, pelvic floor exercises, biofeedback, and functional electrical stimulation; TI - transurethral injection; VAS - visual analog scale; NM - not mentioned; PI - periurethral injection, UTI - urinary tract infection; *p* - *p* value; NS - not statistically significant; VAS - visual analog scale; LPP - leak point pressure; ITT - intention to treat.
